# Understanding perspectives for product design on personal data privacy in internet of things (IoT): A systematic literature review (SLR)

**DOI:** 10.1016/j.heliyon.2024.e30357

**Published:** 2024-04-25

**Authors:** Amparo Coiduras-Sanagustín, Eduardo Manchado-Pérez, César García-Hernández

**Affiliations:** a San Jorge University, Spain; b University of Zaragoza, Spain

**Keywords:** Internet of things, Data privacy, Personal data, User behaviour, Privacy risk, Digital products, Data sharing, Data surveillance, Dataveillance

## Abstract

As the number of Internet users grows, the increase in smart devices interconnected through the Internet of Things (IoT) have contributed to improvements in the functionality of everyday products and enhancement of user experience. Yet, they affect user privacy and render personal data more vulnerable. To foster a digital future fully aware of user privacy requirements, a line of design research emerges that focuses on balancing product innovation with user data protection. This matter relates to sociocultural, economic, and technological aspects, and its core is a human-centered design strategy. Still, there is a gap in academic research oriented towards guiding product developers on how to consider personal data privacy concerns when designing honest IoT devices. To define this gap and delve deeper into this relevant topic, this paper presents a systematic literature review of recent academic research using the Preferred Reporting Items for Systematic Reviews and Meta-Analyses (PRISMA) method. This review focuses on prevalent research topics such as data privacy, personal data, data surveillance, and user behaviour in IoT. The result is a state-of-the-art compilation of 45 scientific studies mapping the most relevant concepts and approaches for product development in the last ten years of research, aligned with some central research questions. The Discussion and Conclusion sections provide a deep understanding of the complexity of the fast-changing landscape of privacy and personal data management using IoT products. Finally, this study proposes future academic research directions devoted to providing product designer specific, specialised help from different (yet interconnected) scientific approaches.

## 1 Introduction

The Cambridge Analytica scandal of 2016 is widely regarded as a pivotal moment in the discourse surrounding the use of personal data by organisations [1]. It revealed the disturbing reality of the sale of citizens' personal data by prominent technology companies to third parties through the use of Internet of Things (IoT) devices and social media platforms (SMPs) [2]. The primary concern raised revolves around the substantial value of personal data, or personally identifiable information (PII), to various entities, including political parties, governments, and markets. The social conflict arising between the ownership and exploitation rights of this valuable data and customers' privacy rights has driven profound reflection and discussion across the political, economic, social, and technological spheres, which ultimately has far-reaching implications for the potential evolution and utilisation of IoT technologies by product developers.

As IoT communication users, we generate a significant amount of data related to our PII, and it is essential to consider how these data are collected, what they are for, and what role developers of such IoT technologies play in this process. Engineers, industrial designers, and software developers are continuously seeking ways to address concerns related to data privacy and security to create fair, transparent, and ethical frameworks. The increasing integration of technology into our daily lives has led to an increasing need to balance the benefits of data-driven innovation by protecting personal privacy and avoiding potential misuse or harm [3]. Thus, it is imperative that ethical considerations and best practices inform the development and deployment of IoT technologies to ensure a safe, secure, and honest digital future.

Unlike the existing literature, the novelty of this study lies in its unique perspective, as it examines the complex issues of privacy, personal data, and IoT technologies not only through an isolated area of software development, informatics, or electronics but also from an integrated perspective of product design practice. Furthermore, this study proposes an additional novel dimension by delving into the user's perspective and enriching the analysis by providing insights into how end users perceive and interact with privacy concerns in the context of IoT technologies. By developing a systematic literature review (SLR), the current study classifies and synthesises recent academic research to propose key trends, patterns, and gaps in the literature. This contributes to a deeper understanding of the relationship between privacy, personal data use, and the development of IoT products and offers valuable insights for future research. In summary, this study makes a significant contribution by providing a novel approach for understanding and addressing personal data privacy concerns through the specialised lens of product design, clearing the way for more ethical and privacy-preserving innovations in the evolving IoT landscape.

The paper is structured as follows: Firstly, in section 1 the introduction of the work done is given, section 2 explains the theoretical evolution of the related terms and topics and section 3 presents the methodology followed for the SLR. Following, the results of the SLR are shown in section 4 and section 5 is for the discussion. Finally, section 6 is for the conclusion of the study.

## 2 Theoretical evolution and proposed framework

This section explores the theories that shaped this research and presents the framework for guiding the analysis. This background examines privacy, personal data, and IoT technologies, focusing on the perspectives of product design and user experience.

### 2.1 Privacy and surveillance

The scientific literature currently focuses on the problem of personal data privacy around IoT technologies, which originates from the concept of surveillance. Historically, the concept of losing control over personal information to someone else has been analysed in the social sciences under the concept of surveillance [4]. The concept of surveillance has evolved with the development of technology. Although there are already some classifications for the different types of surveillance, this study classifies them into three main categories relevant to privacy: physical surveillance, digital surveillance, and networked surveillance (see Table 1).

**Table 1:** Relation between surveillance concepts, years, authors and categorization.

Year	Concept	Work and/or relevant author	Categorization *(P* = *Physical surveillance; D = Digital Surveillance; N* = *Networked Surveillance)*
**1949**	Surveillance	“1984” by George Orwell	P
**1975**	Panopticon	“Panopticon” by Michael Foucault	P
**1988**	Dataveillance	Roger Clarke	D
**1992**	Societies of control	Gilles Deleuze	P, D
**2003**	Social sorting	David Lyon	N
**2004**	Surveillance & cities	David Murakami Wood	N
**2004**	Sousveillance	Steve Mann	D
**2006**	Banopticon	Didier Bigo	N
**2008**	Participatory surveillance	Anders Albrechtslund	D, N
**2012**	Social media surveillance	Daniel Trottier	D, N
**2014**	Digital Sociology	Deborah Lupton	D, N
**2015**	Surveillance Capitalism	Shoshana Zuboff	D, N
**2019**	Digital Subjects	Christian Fuchs	D

#### 2.1.1 Physical surveillance

Physical surveillance refers to monitoring the activities, movements, and interactions of an individual using observations or technology [5]. It involves the use of physical means such as closed-circuit television (CCTV) video cameras, audio recorders, or human observations to gather information about an individual's behaviour, actions, and interactions with others [6]. This type of surveillance focuses on the observation of people, vehicles, or certain activities with a specific reason for investigation [7]. It is also a way of gathering too many facts about a person, who can end up being used for coercion or blackmail [8]. Ubiquitous mass surveillance refers to the surveillance of groups of people, usually large, which targets indiscriminately entire populations [9]. Further, authors such as Schneier [10] and Wood [11] wrote about the digital evolution of physical surveillance infrastructures. An interesting discussion arises about privacy over security because of the common belief in society that impulses fighting terrorism and organised crime [12]. One specific example is the Stasi surveillance implemented by the former German Democratic Republic, which was based on hearing phone taps transcribed manually [13]. Another more recent example is China's Social Credit System (SCS), which processes data to monitor and predict the trustworthiness of citizens and is active for 1.4 billion citizens as of the year 2020.

#### 2.1.2 Digital surveillance

It refers to the monitoring of digital communications and activities of individuals or groups using technology such as computer software, Internet monitoring tools, and data analytics [14]. This type of surveillance involves the interception and analysis of digital data, including phone calls, email and text messages, social media posts, and web browsing activities. In 1988, Clarke [8] defined data surveillance as a new trend or approach to existing surveillance. He refers to this as the conjunction of data and surveillance. He goes further and proposes new policies for the power of technologies and the implications this could have for society. Currently, data surveillance is not simply a descriptive (monitoring) concept but also a predictive (conjecture) and prescriptive (enactment) concept [15]. Degli Esposti [16] described data surveillance, referring to it as the systematic monitoring of people or groups which means that data surveillance involves the methodical tracking of individuals or groups, using PII systems to influence or control their actions. This practice is not isolated but rather intricately linked to two vital components: big data and analytics. Companies rely on interconnected elements to collect and process extensive data, derive insights, and employ them to advance their business goals. Data surveillance is part of a broader ecosystem in which data collection, analysis, and decision-making work together, enabling organisations to make data-informed choices and steer their operations in a direction that aligns with their objectives.

Therefore, our personal digital footprint has high commercial and political value and should be considered a semi-fundamental human right [17]. West [18] explained how tracking technologies in the private sector create an uneven distribution of power, favoring those who can access and utilize personal data. Although the self-regulation efforts of proprietary platforms offer some level of protection, relying on companies that profit from data-driven business models to safeguard their privacy and reputation raises concerns [19].

#### 2.1.3 Networked surveillance

This refers to the use of interconnected devices and systems, such as the IoT, smart products, artificial intelligence (AI)-powered devices, smart cities, and other data-collecting technologies, to monitor and analyse activities which are shared among multiple devices and networks. This type of surveillance allows for the interrelated real-time monitoring and recording of activities [14,18,20]. Shoshana Zuboff in ‘The Age of Surveillance Capitalism: The Fight for a Human Future at the New Frontier of Power’ [21] explains, from a very critical perspective, the new ways of capitalism through companies that are accumulating and using our PII. Specifically, she states that these corporations gather substantial amounts of fresh information from us, but this does not necessarily benefit them. They predict future outcomes for the benefit of others rather than our own. These companies have large amounts of data, knowledge, and surveillance power; therefore, the possible consequences are relevant to the present and future.

IoT surveillance refers to the use of IoT technology to monitor and track people, objects, or environments. It involves the collection of data from connected devices, which can be used to gather information about individuals, their behaviour, and their surroundings and is shared with other devices online and in real time [7]. IoT surveillance can be used in various settings, including smart homes, smart cities, and workplaces, for security, safety, and efficiency. However, concerns have been raised regarding the potential misuse of IoT surveillance, including privacy violations, data breaches, and discrimination. Lupton [22], after deep research about data tracking technologies, draws upon theoretical perspectives on the concepts of selfhood, citizenship, data surveillance, and the global digital data economy. In discussing the wider social and cultural implications of the emergence and development of these modes of self-tracking, the current rise in AI has added a new dimension to these concerns. The services offered by IoT devices, such as SMPs, have also been studied. Social media and the Internet, in general, have generated great change and improved the quality of life of citizens [6,23]. It is also a valid way of working and gaining knowledge, from which great progress has been made [24]. However, there is a feeling of constant surveillance and manipulation associated with the use of SMPs [25].

### 2.2 The challenge of developing IoT products for smart users

In 1982, Carnegie Mellon University pioneered the IoT technology by installing a Coca Cola vending machine connected to the Internet. This innovative device allowed researchers to remotely check the status of the machine and determine whether it was stocked with drinks. Since then, there has been exponential growth in IoT smart computing and digital technologies integrated directly into products [26,27]. Everyday items such as cars, televisions, refrigerators, and others have transitioned from their traditional roles to become IoT-enabled products capable of providing a wide range of services [28] and additionally, they can collect substantial volumes of PII. History of product design of IoT devices was at first characterised by deployment of simple functionalities and limited connectivity [29]. Over the years, advancements in sensor technologies, protocols, chips and wireless communication have driven the evolution of more sophisticated and interconnected products which has shift the IoT product design development field. Designers are now challenged to integrate functionality, aesthetics and user experience while considering the complex network of interactions provided by IoT technologies [30]. This history reflects a continuous journey of innovation, from the rudimentary IoT devices of the past to the intricately designed and interconnected products that define the present landscape of the IoT.

Considering IoT as a network of physical objects, these interconnected objects continuously collect, analyse, and utilize data to trigger action, providing valuable insights for planning, management, and decision-making [31]. Considering that all IoT devices are smart products [4] the current research delves into the conceptualisation of the term smart product, and it is taken as a reference by Mühlhäuser's [32] definition and the one adapted from him by the Smart Products Consortium [33] widely adopted by scholars: ‘A smart product is an autonomous object which is designed for self-organised embedding into different environments in the course of its lifecycle and which allows for a natural product-to-human interaction. Smart products can proactively approach users using the sensing, input, and output capabilities of the environment, thus being self-, situational-, and context-aware. Related knowledge and functionality can be shared by and distributed among multiple smart products and emerge over time’. This implies that smart products and IoT devices collect relevant data not only from users but also from their environments, thereby enabling cross-data acquisition. Innovative product–service system design (PSS), which is enabled by sensor technology, usually relies on remote monitoring, extensive data sharing, and analyses [34], and determines how technology interacts with users [35], including what level of intrusiveness is acceptable.

At the time of writing this paper, the topic of AI is at the core of a profound debate about its possible implications, limits, and potential uses in product design. The European Commission is developing a pioneering regulation called the AI Act, first implemented in 2021, which will clearly guide researchers in the near future [36].

As technologies and their applications to different products have evolved, new user profiles have emerged within a changing social landscape. Users are increasingly aware and conscious of the use of these devices and are more intrigued about their technology, having a higher level of expertise, wider access to knowledge, and heightened demands and expectations [37]. Furthermore, users are now interconnected through SMPs, which not only amplify their awareness of technological trends but also foster a sense of expertise in their personal experiences [38]. Users have transitioned into experts in their own lives, actively engaging with technology and influencing the development of products and services through their insights, preferences, and feedback [39,40]. The relationship between evolving IoT technologies and informed users plays a significant role in our research.

A new network is emerging, characterised by relationships between companies and users, users and products, and users among themselves. User demand for companies and their products has grown, and one of the most developed needs is the demand for honest relationships with both products and companies [41]. In this demand for honesty and reliability, it is discussed the needs and rights of users in relation to the privacy of their PII, which these IoT technologies directly, indirectly, discriminately, and indiscriminately gather, collect, and use from them.

In this study, an SLR was developed to investigate the current state-of-the-art in the scientific field related to privacy in IoT technologies. For this purpose, two research questions were formulated, as shown in Table 2.

**Table 2:** Research questions.

Nº	Research question
RQ1	What are the proposals suggested by researchers in order to address privacy concerns regarding IoT technologies?
RQ2	What is the standing position of users towards their privacy when using IoT technologies?

## 3 Materials and methods

**Fig. 1:**
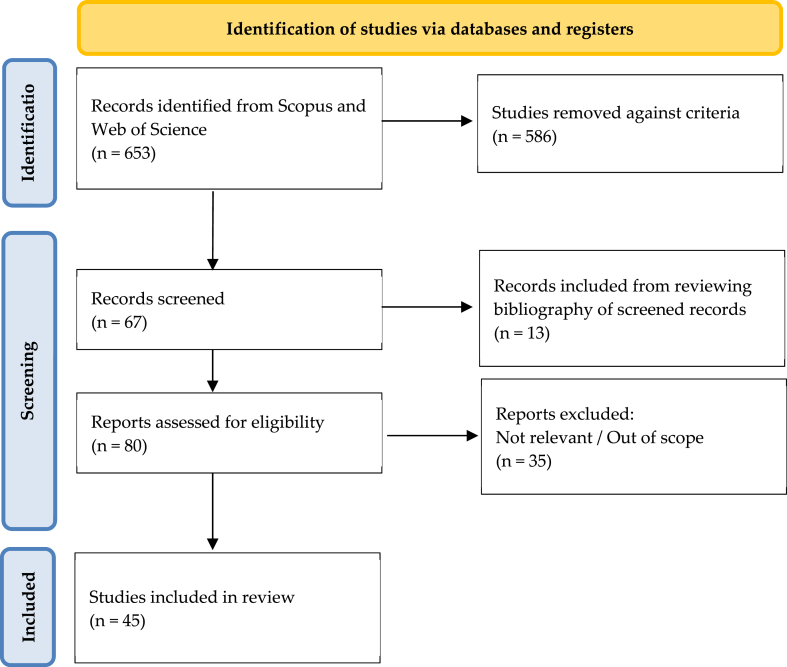
PRISMA flowchart. Source: Created by the authors.

To address the research questions proposed in this study, an SLR was conducted. The present study followed PRISMA [42] (see Fig. 1). The protocol included i) search strategy and database selection, ii) the exclusion criteria, and iii) data extraction and analysis.

### 3.1 Search strategy and database selection

To define the search strategy, the two main research questions have been disaggregated into two essential dimensions: (a) the first one refers to the issue related to the problem under study (“privacy of personal data” OR “data surveillance” OR “personal data use”); (b) the second one refers precisely to the emerging domain in which such lack of privacy materializes ("IoT” OR “Internet of Things” OR “Smart products/technologies"). Then, in order to deepen the current search, two more factors were introduced: (iii) the perspective of the developers and the designers of such IoT products (“IoT development”) and (iv) the user awareness, behaviour and concerns shown by the users of IoT devices (“users' awareness” AND “users’ behaviour”). With all that, the string used for the search was defined and conducted in two distinguished databases: Web of Science and Scopus.

### 3.2 Selection criteria

**Fig. 2:**
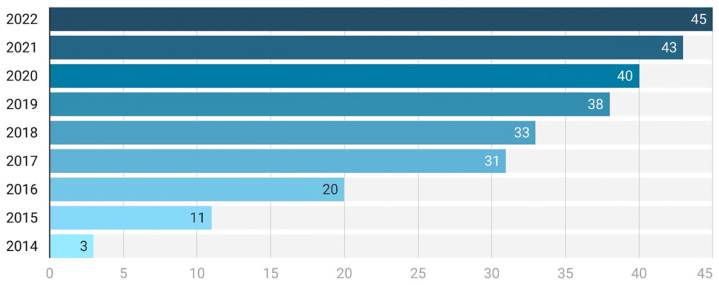
Cumulative number of papers per year.

In order to correctly select the papers to study based primarily on their scientific impact, the inclusion and exclusion criteria was defined. So, the inclusion criteria protocol was: (a) scientific studies focused on the privacy of user's PII in IoT devices, (b) be written in English language, (c) be published in an academic journal or at the proceedings of a high-impact conference; (d) be indexed in Web of Science or Scopus, (e) studies published from 2013 to 2022. On the other hand, the exclusion criteria protocol was: (a) Studies that do not deal with privacy, surveillance and the use of personal data in IoT technologies (b) papers that are not written in English, (c) divulgated studies not published in scientific journals or proceeding of high-impact conferences, (d) papers related to other sciences such as medicine, pharmacology or molecular biology (e) papers published before 2013. It is considered that focusing on research from the past ten years encompasses a wide range of pertinent studies. (see Fig. 2 to see evolution of works).

### 3.3 Data extraction and data synthesis

Theoretical and conceptual frameworks were analysed, as well as empirical studies published in the Science Citation Index (SCI). Additionally, the Social Sciences Citation Index (SSCI) has also been searched for related papers, and following the PRISMA flowchart (see Fig. 1) for the identification of studies, a total of 653 academic works were found. Subsequently, after applying the selection criteria, just 67 studies were screened. Simultaneously, based on the preliminary research, it was found that some relevant studies have been overlooked. It is believed that this is because there is no extended agreement in the terminology and keywords used for this specific subject of the study. Therefore, to identify papers that might have been overlooked during the search, a thorough and in-depth search of the titles, abstracts, and bibliographies of the 67 selected works was done; 13 additional papers were added. Subsequently, a full-text reading was conducted and 35 studies discarded; even though they were included in the first search and were somehow related to the research questions, they did not fit the selection criteria. Finally, the search concluded by selecting 45 studies (see Annex. I).

## 4 Results

This section presents the findings of this study. It describes the key concepts and essential terminology seen in the 45 selected papers and provides insights into the relationship between the concepts under study.

### 4.1 Descriptive analysis of involved research

**Fig. 3:**
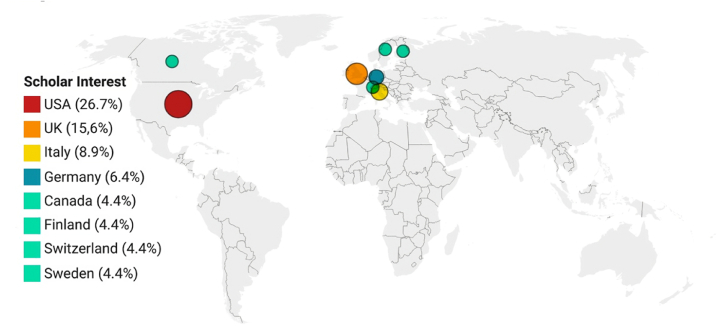
Number of proposals per country.

Once the works for analysis have been selected, it is appropriate to place them in a wider context where relevant information can be identified. Additional context may reveal whether the topic is becoming more popular (see Fig. 2). In this sense, 44.4 % of the proposals were published between 2016 and 2017, and less than 25 % of the proposals were presented between 2010 and 2015. It has also been considered which countries have more academic and scientific research interests. The selected studies originated from institutions in 19 countries (Fig. 3). Most proposals were developed by authors based in the United States of America (USA) (n = 26.7 %), followed by the United Kingdom (UK) (n = 15.6 %) and Italy (n = 8.9 %). The remaining selected studies came from other countries, such as Germany (n = 6.7 %), Canada (n = 4.4 %), Finland (n = 4.4 %), Switzerland (n = 4.4 %), and Sweden (n = 4.4 %). The remaining studies were from France, Greece, India, Japan, Malaysia, the Netherlands, Peru, Portugal, Russia, Saudi Arabia, and Spain, with only one study selected per country. This geographical analysis shows how some countries, such as China, India, Japan, and Korea, which are key players in the use and development of IoT technologies, lack academic or scientific publications on the subject. Regarding the type of publication, almost 63 % of the studies were journal publications, and 37 % were conference publications. Considering the journal indexes in which the selected studies were published, the majority (58 %) came from the Science Citation Index Expanded (SCIE), and only 9 % were from the SSCI. Works published in journals that are indexed in both SCIE and SSCI account for 24 % of the total amount. The remaining 9 % are published in the Emerging Sources Citation Index (ESCI).

### 4.2 Terms and concepts reviewed

**Fig. 4:**
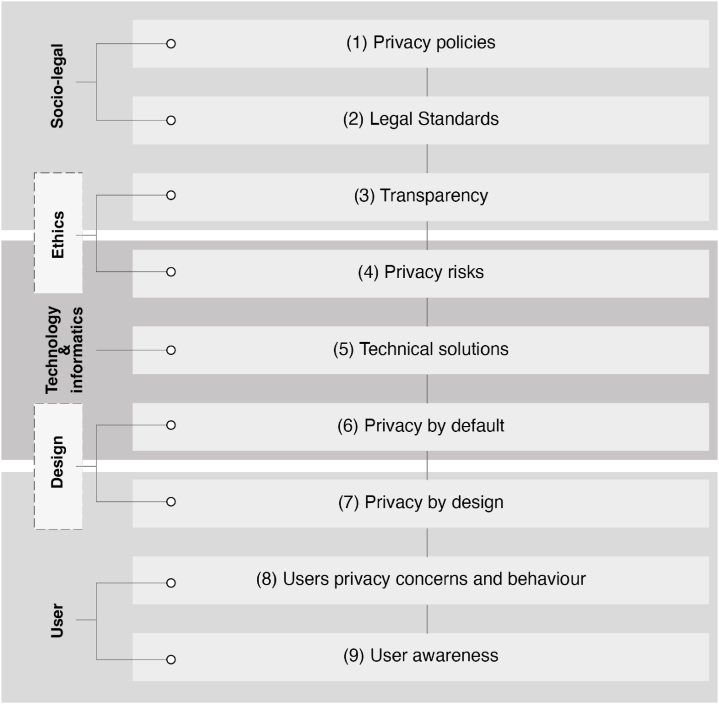
Key concepts found across the various studies examined.

To obtain a thorough understanding of the research landscape related to the selected subject, a rigorous analysis of academic works was conducted. This method allowed the researchers to identify and extract the key concepts that were recurrent across the various studies examined (Fig. 4). These essential themes formed the foundation of our research and served as starting points for the development of our study objectives and research questions. By carefully examining and synthesising these recurring concepts, gaps in the existing literature were found and a unique approach to further investigate the topic was proposed. Overall, this methodological approach allowed us to systematically navigate the vast and complex terrain of existing research and effectively position our study within the context of a larger research landscape.

To ensure clarity and precision, a comprehensive definition of each of these key concepts has been developed. The process of defining each concept involved a thorough review of the relevant literature and an examination of how each concept was used in the studies analysed (see Table 3). These definitions are precise, concise, and aligned with the broader research landscape. These definitions formed an integral part of our research framework and were used to guide our data collection, analysis, and interpretation. They are as follows.

(1) **Privacy policies:** Legal documents detailing how a company handles user data. They outlined the information collected, how it is used and shared, and how users can control their data [43].
(2) **Legal standards such as General Data Protection Regulation (GDPR):** These refer to the legal and ethical standards that govern the collection, processing, and use of personal data in the context of Internet-connected devices, including smart homes and wearables. It emphasises the need for transparency, privacy protection, and alignment with fundamental rights and values [44].
(3) **Protection and Transparency:** It refers to the need for openness and clarity in the collection, processing, and use of PII, as well as the importance of designing technology that aligns with legal and ethical standards. It involves providing users with clear and accessible information about how their data are being used and ensuring that they have control over their data.
(4) **Privacy risks:** These refer to potential threats to individuals' privacy caused by the collection, processing, and use of their personal data in the context of Internet-connected devices and IoT.
(5) **Technical solutions:** The design and implementation of technology must consider social and technical aspects, including legal and ethical standards. These solutions address privacy and security concerns in the context of IoT devices, data collection, and analysis and aim to increase transparency and user awareness.
(6) **Privacy by default:** It means that personal data should be automatically protected without the need for the user to take action [45].
(7) **Privacy by design:** This concept requires privacy considerations to be integrated into the design of products and services, aligned with legal and ethical standards, and customised for business [46].
(8) **Users' privacy concerns and behaviour:** Here, it is referred to how individuals perceive and act regarding their personal information and data online. Users can limit their online activities, use privacy settings, avoid sharing personal information, or be willing to share their data. These issues are complex and are influenced by culture, technology, education, and personal experiences [41].
(9) **User awareness:** Personal data collection and use refer to how much individuals know about the ways in which their personal data are used and shared [47]. It includes knowledge of privacy policies and settings, understanding potential risks, and taking steps to protect online data. Awareness can be influenced by education, media coverage, and personal experience.

**Table 3:** Relation of the key concepts found in the analysed studies.

#	PRIVACY				DESIGN AND DEVELOPMENT			USER	
	Privacy policies	Legal Standards (GDPR,..)	Protection and transparency	Privacy risks	Technical solutions	Privacy by default	Privacy by design	Users privacy concerns and behaviour	Users awareness
1	✓	✓	✓	✓					✓
2				✓				✓	✓
3							✓	✓	✓
4		✓	✓						
5				✓		✓	✓	✓	
6	✓				✓				✓
7			✓			✓	✓	✓	
8							✓	✓	✓
9					✓			✓	✓
10				✓	✓			✓	✓
11				✓				✓	✓
12								✓	✓
13	✓			✓				✓	✓
14								✓	✓
15	✓		✓	✓			✓	✓	✓
16			✓		✓		✓	✓	✓
17	✓						✓	✓	✓
18	✓		✓	✓					
19				✓				✓	✓
20	✓		✓	✓	✓				✓
21	✓	✓		✓	✓			✓	✓
22	✓	✓	✓		✓		✓	✓	✓
23					✓		✓	✓	✓
24	✓			✓	✓		✓	✓	✓
25				✓	✓				
26				✓	✓			✓	✓
27	✓			✓	✓	✓	✓	✓	✓
28		✓	✓		✓	✓	✓		
29	✓	✓		✓				✓	✓
30							✓	✓	✓
31	✓				✓				✓
32				✓	✓		✓	✓	✓
33				✓	✓				✓
34					✓			✓	✓
35					✓			✓	
36					✓				
37					✓				
38	✓				✓			✓	
39									
40	✓			✓	✓				✓
41	✓				✓			✓	
42	✓				✓				
43	✓			✓					
44	✓			✓	✓		✓		
45	✓			✓	✓		✓	✓	

After placing the proposed solutions in specific contexts, the paper focuses on responding to the previously raised research questions. The following sections of the document detail the relevant components and present the findings in both written and visual formats.

## 5 Discussion

The purpose of this SLR was to identify and synthesise key aspects and concepts that the academic world has studied on the topic of privacy in IoT devices. In this section, it is summarised the main findings of this review and the specific research questions are answered.

### 5.1 Answering research question 1

Regarding RQ1, what are the proposals suggested by researchers in order to address privacy concerns regarding IoT technologies? Researchers have studied several possibilities. Two main categories have been identified in which the proposals offered by scholars are grouped: (A) regulated systems: legal measures and (B) technical proposals, which have two other categories: (B.1) propositions from the design and development practice and (B.2) technological strategies.

(A) Regulated system: Legal measures

Many authors have proposed that to protect user privacy in the IoT, there must be a consistent legal regulation that applies to all organisations handling personal data. One of the most relevant international regulations is the GDPR, published in May 2018 by the European Union. Garcia et al. [48] explain how the GDPR mandates the fulfilment of a specific legal basis for the processing of PII, aligning with the core principles of data protection law. Prior to that, in 2017, McDermott [49] proposed a new fundamental right to data protection under the Charter of Fundamental Rights of the European Union, in which he underlined values and implementation challenges and emphasised the critical need to adapt legal strategies to address the intricate interplay between privacy rights and technological advancements. Many authors, such as Bernal [50] and Monreale et al. [49], delved into the foundational principles of privacy and data protection, as well as the complex balance required between safeguarding individual autonomy, transparency, preventing discrimination, and the regular and extended use of these kinds of technologies. They all suggested recasting the debate to raise the bar for the justification of surveillance and finding a new balance in political debate, law, and decision-making.

Others, such as Kuznetsov et al. [43], Chennamaneni and Gupta [51], and Garcia et al. [48], are also concerned with privacy policies, data collection, and data protection laws in the context of Internet-connected devices. Hauser et al. [47] discussed ethical issues arising from the increasing complexity of technology. Saura et al. [45] were concerned with privacy issues in the context of data sharing and analysis in the Social Internet of Things (SIoT). Overall, the authors discussed the need for more transparency in the collection, processing, and use of personal data, as well as the importance of designing technology that is aligned with legal and ethical standards.

(B) Technical proposals

This section presents proposals emerging from both design and development practices and various technological strategies aimed at addressing privacy concerns in the context of IoT technologies.

(B.1.) Propositions from the design and development practice

Some researchers suggest that, rather than relying solely on legal measures, there is a growing need to take a different approach to ensuring the privacy of PII within the context of IoT technologies. This approach involves actively controlling and managing privacy concerns during the design and development of IoT devices. This can be achieved through close collaboration among those responsible for creating and shaping IoT devices, including developers, designers, and final IoT users.

Cavoukian [46] first introduced the concept of Privacy by Design (PbD) in the 1990s as a response to emerging privacy concerns resulting from the growing use of technology and the Internet. PbD is now recognised as an international standard and has been incorporated into laws and regulations in various jurisdictions worldwide. Even the GDPR includes guidelines concerning PbD, in which it is dictated that developers must guarantee, through technical and organisational measures, their adherence to the fundamental principles of data protection law, as outlined in Article 25 of the GDPR [48]. This concept emphasises the need to prioritise privacy and data protection at every stage of the design and development of new technologies, products, and services. This means incorporating privacy and data protection features into the initial design rather than treating them as an afterthought or add-on. Furthermore, in the current research reviewed, she explained how PbD can be practically implemented and customised by businesses to proactively embed privacy into the design of IoT products through the seven foundational principles of PbD.

Additionally, it is important to ensure that the collected data are used for the intended purposes and are not misused or abused. Monreale et al. [52] discussed the application of the PbD principle to big data analytics to protect user privacy. It proposes a schema for guaranteeing user privacy protection, such as k-anonymity, microaggregation, and privacy-preserving distributed-movement data aggregation. The PbD principle incorporates privacy protection into knowledge discovery technology. This means that the data analysis considers relevant privacy requirements.

McDermott [49], Schaub et al. [53], Torre and Koceva [54], Ando et al. [55], Libaque-Saenz et al. [56], Custers [57], and Montanari et al. [58] discussed the importance of privacy protection at the design level. They proposed various principles for designing effective privacy notices and controls and suggested the use of expiry dates for consent as a tool for addressing some of the issues related to informed consent. The studies analysed observed the main challenges and opportunities for designing IoT devices that comply with legal and ethical standards and ensure transparency and privacy protection in data collection, processing, and use. They have various ideas surrounding how user experience (UX) should be considered and designed under the umbrella of data protection in the context of IoT.

Hauser et al. [47], Saleem et al. [26], Pillan et al. [59], and Worthy et al. [60] discussed the ethical and philosophical issues arising from the increasing complexity of technology. They highlighted the need to consider privacy and control issues, the results of design decisions regarding the complex aspects of UX, and the significance of building trust with IoT owners. Furthermore, in a study conducted by Hernández-Ramírez in 2019, it was highlighted that when designing AI-powered products, it is crucial to emphasise the human and socio-technical aspects that come into play when interacting with IoT technologies, with trust being identified as a critical factor. They explored ways in which IoT systems could be structured to enhance trust among users. Schaub et al. [53] evaluated the inefficacy of current privacy notifications and settings while deliberating on the principles for designing more effective privacy notices and settings. Pillan et al. [59] examined the impact of design choices on the complex aspects of UX, including self-perception, privacy, and personal identity.

(B.2.) Technological strategies

Das et al. [61] discussed the adoption of computer vision-based technologies and their potential privacy concerns and proposed a distributed privacy infrastructure for IoT to enhance user awareness and control over the collection and use of video data. Matzner [62] argues that the advent of ubiquitous computing and the prevalence of big data necessitate the development of novel paradigms and mechanisms for the protection of individual privacy. Even when people act in full conformity with privacy norms, they can infringe on the privacy of others. The studied paper reviews Helen Nissenbaum's [63] ‘privacy as contextual integrity’ concept and proposes a new notion, the reduction of data, particularly in terms of data quality and potential promising technical enhancements for privacy. In addition, Montanari et al. [58] conducted a qualitative study on user privacy concerns regarding connected objects, focusing on data ownership, and provided guidelines for designing an intuitive tool to address this concern. Similarly, Aleisa and Renaud [64] discussed the privacy concerns associated with IoT and the lack of privacy-preserving solutions that satisfy core privacy principles. They suggested future research based on the assumption that end users are willing to expend effort to protect their privacy. Sicari et al. [65] outlined the principal research obstacles and existing solutions in the field of IoT security, identified open issues, and offered potential directions for future research.

Rawassizadeh et al. [66] presented a synthesis of technical and user-focused discoveries derived from three lifelogging studies and quantified self-data collection studies conducted in real-life scenarios involving smartphones and smartwatches. The use of sensor-rich wearable devices and smartphones has led to an increase in lifelogging and quantified self-fields. This study highlights the need to minimise manual intervention, consider data loss and uncertainty, and address the lack of multivariate reflection methods. Researchers should consider these challenges and adopt solutions such as removing manual user interactions, data analysis and mining, and on-device data analysis. Similarly, Torre and Koceva [54] present a framework for managing privacy protection in personal IoT devices that integrates personal data managers and inference prevention techniques. Finally, Custers [57] highlighted issues with informed consent for processing PII, and suggested the use of expiry dates as a tool to address some of these issues.

Rehman et al. [38] presented a personal data ecosystem in which smartphones and wearable devices were used as data-mining platforms in ubiquitous, pervasive, and big data environments. They conducted an extensive review of the recent literature, presented a detailed taxonomy, evaluated performance metrics, and highlighted future research directions and new ways and contexts in which personal data mining can be used. Van de Pas and van Bussel [67] suggested that addressing citizens' privacy concerns goes beyond technological privacy compliance and should incorporate a conceptual model to enable privacy impact assessments. Weber [68] highlighted the growing need for regulatory and technical actions to protect individual privacy in the IoT context. Perera et al. [69] suggested that user privacy must be protected throughout the entire data management lifecycle of IoT solutions and called for strict laws and regulations, as well as the development of privacy-preserving algorithms. Additionally, Saleem et al. [26] discussed the hardware and software challenges related to the creation and acceptance of immersive and wearable technologies embedded with IoT, highlighting bottlenecks and proposing guidelines for applications in SMPs, healthcare, and banking. In addition, Adams [70] discussed the privacy challenges associated with big data and IoT, highlighting the need for individual privacy protection in the collection, storage, sharing, and analysis of data.

Finally, Ando et al. [55] proposed employing statistical analysis to study how users perceive privacy and security risks in IoT. Countermeasures such as data encryption, legal structures, and hardware have been suggested; however, additional survey analysis is required to identify effective measures. Service providers must consider the findings of the analysis and implement suitable measures to reduce user concerns about privacy and security risks in IoT services. Porambage et al. [71] discuss the technological aspects of IoT privacy issues and challenges from the viewpoints of users, datasets, underlying technologies, and relevant legal regulations. It also examines existing solutions and emerging approaches, such as PbD, and open research issues for preserving privacy in IoT.

Product conceptualisation is the core and key to providing honest management of PII based on agreements between product providers and users, and thus constitutes the focus of this study. Nevertheless, it would not be enough if this information were accessed by malicious agents that pretended to misuse this PII. Therefore, it seems necessary to briefly underline that in parallel with the development of honest products from a conceptual perspective, the development of certain privacy protection technologies makes a strong contribution to safeguarding user data and information from third-party assaults. Privacy protection in the IoT ecosystem is undergoing a paradigm shift with the emergence of technologies such as post-quantum cryptography (PQC), but this is not limited to Ref. [72]. Recognising the potential of PQC, it is imperative to consider their influence on security applications ranging from smartphones to blockchains [73]. Furthermore, there are studies on the implications of blockchain technologies, privacy, and security management of personal data [74]. In addition, key works such as studies on lightweight hardware architectures and error detection schemes [75,76] delve into fault diagnosis which is an essential aspect for ensuring cryptographic robustness. Moreover, insights from studies on side-channel attacks and lightweight cryptography or PQC contribute to a holistic understanding of the security challenges in the IoT landscape [77]. The finalisation of the NIST lightweight standardisation in February 2023 signifies a crucial milestone for IoT security. However, to address privacy concerns comprehensively, it is essential to explore fault attacks as side-channel attacks, focusing on error detection mechanisms. This integrated approach ensures a strong foundation for security and privacy in the evolving IoT industry, and is a relevant topic for future studies.

### 5.2 Answering research question 2

In addressing RQ2, what is the standing position of users towards their privacy when using IoT technology? The attitudes that users adopt concerning their privacy while engaging in IoT technologies is examined. This investigation was fundamental in uncovering the attitudes, concerns, and behaviours exhibited by users when using the IoT. By examining the selected studies, valuable insights into the complex interactions between individuals and their privacy expectations was gained. This exploration not only sheds light on the current landscape but also paves the way for informed discussions and recommendations aimed at addressing user privacy concerns effectively.

(A) Privacy paradox and users' inconsistency

From the selected studies, an important contradiction in user behaviour was observed, known as the Privacy Paradox. It was defined by Kokolakis [78] as the inconsistency between privacy attitudes and behaviours, in which many different factors are present, and the same author suggested the need for further research to better understand it. Rohunen and Markkula [79] emphasised that users perceive the privacy risks associated with IoT technology in various ways. Generally, they are concerned about who can access their data, to whom their data are disclosed, and the potential repercussions arising from the various uses of their personal information. To further illustrate these concerns, the focus is set on the study by Malkin et al. [80] which surveyed U.S. Internet users to appreciate their expectations and understanding of how smart TVs collect and use data, finding a wide range of opinions and assumptions as well as an agreement among users that sharing data is unacceptable. However, Zheng et al. [81] conducted 11 interviews with smart homeowners and found that convenience and connectedness dictated users' privacy-related behaviours when dealing with external entities. It is concluded that users trust IoT device manufacturers to protect their privacy and that users lack awareness of privacy risks from inference algorithms working with their data.

**Fig. 5:**
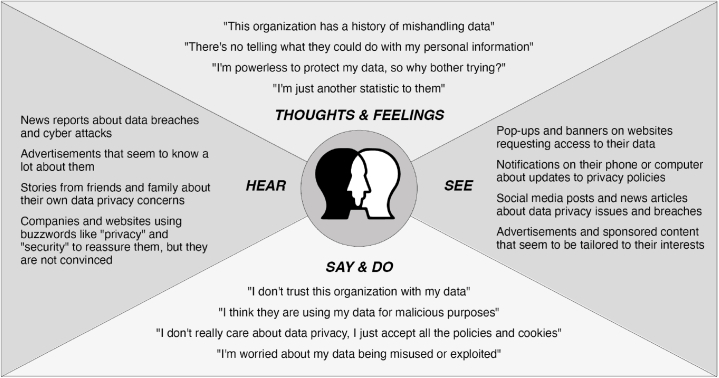
Empathy map showing users' inconsistency.

Similarly, Aleisa and Renaud [64] conducted a study in Saudi Arabia to assess the privacy implications of IoT devices and found that users often ignore potential privacy invasions because of their need for convenience. Functionality was considered the most important factor in purchasing decisions, whereas privacy and security were secondary considerations. In addition, Williams et al. [82] explored the Privacy Paradox in the IoT and found that it is even more prevalent owing to a lack of awareness. Fig. 5 presents a synthesis of how users relate to this phenomenon.

(B) Social context and other factors influencing users' attitudes and behaviour

Social context is a determinant of users' attitudes and an important antecedent to users' behaviour. Rohunen and Markkula [79] described the fact that some factors influence users' attitudes and behaviours towards data sharing, collection, and usage, including how much users are informed about privacy-related news and conversations in public, the impacts of recent privacy breaches, and their adjustment to a culture that involves inclusive data collection. Additionally, people's limited rationality when making decisions and misunderstandings regarding the pros and cons of sharing data, social norms, emotions, and cognitive shortcuts can impact their attitudes and actions. Similarly, Emami-Naeini et al. [83] conducted a study on privacy expectations and preferences in 380 IoT data collection and use scenarios. Their findings suggest that privacy preferences are diverse and context dependent. Another influential factor is explained by Libaque-Saenz et al. [56] who discussed how privacy concerns limit the use of customer data for secondary purposes and suggested investigating how information practices affect the intention to permit the secondary utilisation of such PII.

Prior research has suggested that the way organisations handle information can impact individuals' intentions when deciding whether to participate in situations where their personal data are collected and used (referred to as privacy-related behavioural intentions). From the perspective of procedural fairness, the Federal Trade Commission (FTC) has suggested that information practices encompass five key principles: enforcement, access, choice, security, and notice [56]. However, given the context and determinants identified, some authors have observed that users show some resistance to the use of IoT, as in the case of Mani and Chouk [84] who conducted a study on why consumers might resist using smart services from a privacy standpoint. It was discovered that issues related to information privacy, the unauthorised secondary use of personal data, and feelings of intrusion influenced consumer resistance to IoT and smart services. These concerns are often influenced by the "Big Brother” effect, which is mainly understood as a personal feeling of thinking that our whole lives are under constant surveillance and that digital companies closely observe our personal data [84].

Finally, Tabassum et al. [85] conducted a study with smart home users to understand their knowledge, perceptions, and concerns regarding smart home device data collection, sharing, and usage practices and provided recommendations for policymakers, researchers, and designers. The recommendations are as follows: (1) enhance transparency and control; (2) develop best practices for companies and users; (3) develop mechanisms to increase user awareness about visual indicators and controls; and (4) educate people about future risks.

## 6 Conclusions

This section presents the main and final conclusions of the study and its limitations. It also reflects on and explains the possible future research directions that have emerged as a result of this study.

### 6.1 Practical implications: opportunities for design and development fields

The current literature on privacy related to IoT technologies indicates that significant challenges must be addressed. Similarly, linked to the design and development of IoT devices, there are opportunities to strengthen the privacy protection of IoT technology users.

The research highlights a significant gap in the areas of data privacy and personal data handling within IoT technologies: the lack of a standardised language corpus and clear terminology complementary to the legal and normative structure. This deficiency poses a significant challenge, making it difficult for scholars, policymakers, and industry experts to communicate and collaborate. The lack of a unified vocabulary complicates the formulation of cohesive frameworks and guidelines, impeding advancements in ensuring user privacy in the IoT context. An emphasis is placed into the urgent necessity for the development of a standardised language body that defines key terms and concepts, which would not only make scholarly discussions clearer but also provide a solid resource for practitioners, helping them better navigate the complexities of data privacy.

Through the SLR, a significant gap has also been identified in the territory of IoT device industrial design, pointing to the scarcity of studies addressing personal data privacy from a design perspective. This underlines the urgent need for more in-depth research in this field, recognising the pivotal role that design and designers can play in shaping both user experiences and privacy considerations. Additionally, the analysis concluded that insufficient designer involvement was available from the inception of IoT product ideation. By integrating design perspectives early in the product development process, it is possible to bridge these critical gaps and provide a method for user-centric, privacy-aware IoT technologies. It was also concluded that criticism of social science in addressing data privacy is crucial; consequently, there should be greater scientific permeability among purely technological approaches, industrial design, product developers, and social scientists.

The study demonstrates that users are aware of the existing privacy risks related to IoT technologies but that their behaviors vary depending on the situations in which they are presented. In terms of trusting, sharing, and using these devices, behavior is influenced by various factors. These factors include the social context, trust in the device and the company behind it, perception of benefits from using the device, and level of knowledge and understanding of technology. Additionally, this study shows that many users are willing to share their personal information if they believe it will improve their experience or if they trust that the company will manage their PII responsibly. Thus, the research concludes that user behaviour is more complex than a mere choice between sacrificing privacy to access a service or prioritizing privacy and abstaining from it, as it essentially depends on the user's context and preferences.

To ensure an appropriate balance between data privacy demands and functional needs, software developers, industrial designers, and engineers must foster interdisciplinary dialogues with legal experts. To generate a new era of privacy-aware IoT devices, professionals, policymakers, and developers must collaborate at every stage of the design and development process of such devices to ensure that they comply with legal and ethical standards and guarantee data privacy protection. Based on these premises, a new design and development methodology emerges from UX research, which leads to the opportunity to incorporate participatory approaches that allow users to contribute to and influence the design of such IoT devices, giving users the possibility of improving their sense of personal data handling, privacy, and security. In addition, participatory approaches can guide the creation of privacy design heuristics, privacy-aware solutions, and new ways to incorporate these concepts into human-computer interactions.

These findings have essential practical implications for creating solutions that strengthen privacy protection. The importance of continuous research and constant collaboration with different experts in this area is also highlighted, with the aim of effectively addressing these challenges and adapting to an environment that is constantly changing.

### 6.2 Limitations

Similar to other review papers, the present systematic review was limited by the search string and terms involved, the databases accessed, and the specific time period of the papers included. It is important to note that the paper selection process for this review introduces a risk of bias. To mitigate this, transparent guidelines for exclusion and inclusion criteria will contribute to a stronger and more objective selection process. There is an additional limitation associated with the expertise of the researchers. As product design engineers, the depth of analysis of aspects such as legal issues related to specific topics could be limited. This is a limitation that could influence the overall interpretation of the findings. Efforts have been made to moderate this limitation, including consulting legal experts where possible; however, this remains a factor worth considering in the broader context of the review's scope and depth. Overall, the rapid evolution of the topics considered makes this study valuable, and it will be updated in the near future.

### 6.3 Future research lines

In light of the complexities revealed in our study, it is evident that standardised and universally accepted terminology is imperative, which is why we advocate the establishment of an interdisciplinary and international glossary/protocol that unifies terminology in the domain of IoT privacy. This collaborative effort among technologists, legal experts, policymakers, designers, developers, and researchers will bridge communication gaps, ensuring that conversations about privacy in IoT technologies are coherent and meaningful.

The potential of new research on the influence of UX design and its graphical translation as a user interface (UI) in users’ comprehension of personal data privacy in IoT devices is also acknowledged. This can be interesting from the perspective of determining whether such a design acts as a facilitator or an impediment to how users understand the privacy-related aspects of IoT technologies.

Moreover, the evolving landscape of AI languages and technologies introduces a new frontier in privacy. Exploring the direct impact of AI advancements on privacy in IoT is crucial for future research. Understanding how emerging AI technologies shape data privacy within IoT devices is essential for developing effective safeguards and policies in an ever-evolving technological landscape.

## Funding statement

This research did not receive any specific grant from funding agencies in the public, commercial, or not-for-profit sectors.

## Data availability statement

Data included in article/supp. material/referenced in article.

## Additional information

No additional information is available for this paper.

## CRediT authorship contribution statement

**Amparo Coiduras-Sanagustín:** Writing – review & editing, Writing – original draft, Visualization, Validation, Methodology, Investigation, Data curation, Conceptualization. **Eduardo Manchado-Pérez:** Writing – review & editing, Writing – original draft, Supervision, Methodology, Investigation. **César García-Hernández:** Writing – review & editing, Writing – original draft, Supervision, Methodology, Investigation.

## Declaration of competing interest

The authors declare that they have no known competing financial interests or personal relationships that could have appeared to influence the work reported in this paper.

## Figures and Tables

**Table:** 

Number of the study	Authors	Title	Journal
1	(Kuznetsov et al., 2022)	Privacy Policies of IoT Devices: Collection and Analysis	MDPI Sensors
2	(Chennamaneni and Gupta 2022)	The privacy protection behaviours of the mobile app users: exploring the role of neuroticism and protection motivation theory	BIT Journal
3	(Hauser, Redström, and Wiltse 2021)	The widening rift between aesthetics and ethics in the design of computational things	AI & SOCIETY
4	(Garcia et al., 2021)	Towards Privacy-Friendly Smart Products	18th International Conference on Privacy, Security and Trust (PST)
5	(Saura, Ribeiro-Soriano, and Palacios-Marqués 2021)	Setting Privacy “by Default” in Social IoT: Theorizing the Challenges and Directions in Big Data Research	Big Data Research
6	(George et al., 2020)	Integrating social and technical solutions to address privacy in smart homes.	Systems and Information Engineering Design Symposium (SIEDS)
7	(Cavoukian 2020)	Understanding How to Implement Privacy by Design, One Step at a Time	IEEE CONSUMER ELECTRONICS
8	(Hernández-Ramírez 2019)	On false augmented agency and what surveillance capitalism and user centered design have to do with it	JOURNAL OF SCIENCE AND TECHNOLOGY OF THE ARTS
9	(Rohunen and Markkula 2019)	On the road – listening to data subjects' personal mobility data privacy concerns	BIT Journal
10	(Tabassum, Kosinski, and Lipford 2019)	“I don't own the data”: End User Perceptions of Smart Home Device Data Practices and Risks	Proceedings of the Fifteenth Symposium on Useable Privacy and Security.
11	(Mani and Chouk 2019)	Impact of privacy concerns on resistance to smart services: does the 'Big Brother effect' matter?	JOURNAL OF MARKETING MANAGEMENT
12	(Yao et al., 2019)	Ubiquitous Privacy: Research and Design for Mobile and IoT Platforms	Conference Companion Publication of the 2019 on the Computer Supported Cooperative Work and Social Computing
13	(Malkin et al., 2018)	“What Can't Data Be Used For?” Privacy Expectations about Smart TVs in the U.S.	Proceedings of the 3rd European Workshop on Useable Security (EuroUSEC)
14	(Zheng et al., 2018).	User Perceptions of Smart Home IoT Privacy	Proceedings of the ACM on human-computer interaction,
15	(Saleem et al., 2017)	Design and deployment challenges in immersive and wearables technologies	BIT Journal
16	(Pillan, Varisco, and Bertolo 2017)	Facing Digital Dystopias: A Discussion about Responsibility in the Design of Smart Products	Proceedings of the Conference on Design and Semantics of Form and Movement
17	(Adams 2017)	Big Data and Individual Privacy in the Age of the Internet of Things	TECHNOLOGY INNOVATION MANAGEMENT REVIEW
18	(McDermott 2017)	Conceptualising the right to data protection in an era of Big Data	Big Data & Society
19	(Kokolakis 2017)	Privacy attitudes and privacy behaviour: A review of current research on the privacy paradox phenomenon	Computers & Security
20	(Aleisa and Renaud 2017)	Yes, I know this IoT Device Might Invade my Privacy, but I Love it Anyway! A Study of Saudi Arabian Perceptions	2nd International Conference on Internet of Things, Big Data and Security (IoTBDS)
21	(Das et al., 2017)	Assisting Users in a World Full of Cameras. A Privacy-aware Infrastructure for Computer Vision Applications	IEEE Conference on Computer Vision and Pattern Recognition Workshops
22	(Schaub, Balebako, and Cranor 2017)	Designing Effective Privacy Notices and Controls	IEEE Internet Computing
23	(Emami-Naeini et al., 2017)	Privacy expectations and preferences in an {IoT} world.	13 Symposium on Useable Privacy and Security (SOUPS 2017)
24	(Williams, Nurse, and Creese 2017)	Privacy is the boring bit: user perceptions and behaviour in the internet-of-things.	15th Annual Conference on Privacy, Security and Trust (PST)
25	(Torre and Koceva 2017)	A framework for personal data protection in the IoT	11th International Conference for Internet Technology and Secured Transactions (ICITST)
26	(Ando, Shima, and Takemura 2016)	Analysis of Privacy and Security Affecting the Intention of Use in Personal Data Collection in an IoT Environment	IEICE Transactions on Information and Systems
27	(Libaque-Saenz et al., 2016)	The role of perceived information practices on consumers' intention to authorise secondary use of personal data	BIT Journal
28	(Porambage et al., 2016)	The Quest for Privacy in the Internet of Things	IEEE Cloud Computing
29	(Custers 2016)	Click here to consent forever: Expiry dates for informed consent	Big Data & Society
30	(Montanari et al., 2016)	Understanding the Privacy Design Space for Personal Connected Objects	Proceedings of the 30th International BCS Human Computer Interaction Conference 30
31	(Aleisa and Renaud, 2016)	Privacy of the Internet of Things: A Systematic Literature Review	50th Annual Hawaii International Conference on System Sciences(HICSS)
32	(Worthy, Matthews, and Viller 2016)	Trust me: doubts and concerns living with the Internet of Things.	Proceedings of the 2016 ACM Conference on Designing Interactive Systems
33	(Bugeja, Jacobsson, and Davidsson 2016)	On Privacy and Security Challenges in Smart Connected Homes	European Intelligence and Security Informatics Conference
34	(Bernal 2016)	Data gathering, surveillance and human rights: recasting the debate	Journal of Cyber Policy
35	(Rawassizadeh et al., 2015)	Lesson Learned from Collecting Quantified Self Information via Mobile and Wearable Devices	Journal of Sensor and Actuator Networks
36	(Rehman et al., 2015)	Mining Personal Data Using Smartphones and Wearable Devices: A Survey	MDPI Sensors
37	(van de Pas and van Bussel 2015)	‘Privacy Lost - and Found?’ The information value chain as a model to meet citizens' concerns	The Electronic Journal Information Systems Evaluation
38	(Weber 2015)	Internet of things: Privacy issues revisited	Computer Law & Security Review
39	(Yan, Lu, and Zhang 2015)	Privacy disclosure from wearable devices	Proceedings of the 2015 Workshop on Privacy-Aware Mobile Computing.
40	(Perera et al., 2015)	Big Data Privacy in the Internet of Things Era	IT Professional
41	(Punagin and Arya 2015)	Privacy in the age of Pervasive Internet and Big Data Analytics – Challenges and Opportunities	International Journal of Modern Education and Computer Science
42	(Sicari et al., 2015)	Security, privacy and trust in Internet of Things: The road ahead.	Computer Networks
43	(Matzner 2014)	Why privacy is not enough privacy in the context of “ubiquitous computing” and “big data”	Journal of Information, Communication and Ethics in Society
44	(Monreale et al., 2014).	Privacy-by-design in big data analytics and social mining	EPJ Data Science
45	(Ziegeldorf, Morchon, and Wehrle 2014)	Privacy in the Internet of Things: Threats and Challenges	Security and Communication Networks
