# Corrigendum to “Understanding perspectives for product design on personal data privacy in internet of things (IoT): A systematic literature review (SLR)” [Heliyon Volume 10, Issue 9, May 2024, Article e30357]

**DOI:** 10.1016/j.heliyon.2025.e43310

**Published:** 2025-04-10

**Authors:** Amparo Coiduras-Sanagustín, Eduardo Manchado-Pérez, César García-Hernández

**Affiliations:** a San Jorge University, Spain; b University of Zaragoza, Spain

In this article, the reference (Hernández-Ramírez 2019) is featured in Annex 1 but was not included in the reference list.

The correct version of the reference should be as below:

*[86] Hernández-Ramírez, C. (2019). On false augmented agency and what surveillance capitalism and user centered design have to do with it. Journal of Science and Technology of the Arts, 11(2), 72–81*.

The authors apologize for the errors.

## Declaration of competing interest

The authors declare that they have no known competing financial interests or personal relationships that could have appeared to influence the work reported in this paper.

